# Artificial Intelligence Based COVID-19 Detection and Classification Model on Chest X-ray Images

**DOI:** 10.3390/healthcare11091204

**Published:** 2023-04-22

**Authors:** Turki Althaqafi, Abdullah S. AL-Malaise AL-Ghamdi, Mahmoud Ragab

**Affiliations:** 1 Information Systems Department, HECI School, Dar Al-Hekma University, Jeddah 34801, Saudi Arabia; 2 Information Systems Department, Faculty of Computing and Information Technology, King Abdulaziz University, Jeddah 21589, Saudi Arabia; 3 Information Technology Department, Faculty of Computing and Information Technology, King Abdulaziz University, Jeddah 21589, Saudi Arabia; 4 Mathematics Department, Faculty of Science, Al-Azhar University, Naser City 11884, Cairo, Egypt

**Keywords:** COVID-19 diagnosis, deep learning, chest X-rays, quantum neural network, sine cosine algorithm

## Abstract

Diagnostic and predictive models of disease have been growing rapidly due to developments in the field of healthcare. Accurate and early diagnosis of COVID-19 is an underlying process for controlling the spread of this deadly disease and its death rates. The chest radiology (CT) scan is an effective device for the diagnosis and earlier management of COVID-19, meanwhile, the virus mainly targets the respiratory system. Chest X-ray (CXR) images are extremely helpful in the effective diagnosis of COVID-19 due to their rapid outcomes, cost-effectiveness, and availability. Although the radiological image-based diagnosis method seems faster and accomplishes a better recognition rate in the early phase of the epidemic, it requires healthcare experts to interpret the images. Thus, Artificial Intelligence (AI) technologies, such as the deep learning (DL) model, play an integral part in developing automated diagnosis process using CXR images. Therefore, this study designs a sine cosine optimization with DL-based disease detection and classification (SCODL-DDC) for COVID-19 on CXR images. The proposed SCODL-DDC technique examines the CXR images to identify and classify the occurrence of COVID-19. In particular, the SCODL-DDC technique uses the EfficientNet model for feature vector generation, and its hyperparameters can be adjusted by the SCO algorithm. Furthermore, the quantum neural network (QNN) model can be employed for an accurate COVID-19 classification process. Finally, the equilibrium optimizer (EO) is exploited for optimum parameter selection of the QNN model, showing the novelty of the work. The experimental results of the SCODL-DDC method exhibit the superior performance of the SCODL-DDC technique over other approaches.

## 1. Introduction

COVID-19 revolutionized the healthcare system worldwide. Along with global economics, healthcare, transportation, and education have all been changed [1]. This disease may lead to severe respiratory sickness, but it can be healed with proper treatment. However, human-to-human interaction and community proliferation are the most dangerous side effects of the virus. In cluster cases, a prediction-based artificial intelligence (AI) can identify cases using these methods [2]. In addition, prior medical data are used for making the healthcare data prediction. AI involves a set of algorithms and mathematical models that are designed to simulate human intelligence. Moreover, AI can understand and describe the progression of the COVID-19 vaccine [3,4]. The current patient screening, tracking, predicting, and analyzing must be conducted to accurately predict COVID-19 cases that might assist in the prediction of infected persons in the future [5,6]. Now, AI is often used to find novel compounds to help combat COVID-19. Substantial research has been performed to find new treatments to cure the disease [7], along with computational methods to identify infected persons using medicinal image processing of X-ray pictures and CT scans.

In recent years, Convolutional Neural Networks (CNN) have become the most conventional technique in AI. CNN was effectively applied in medical image analyses such as ultrasonography, magnetic resonance imaging (MRI), X-rays, computed tomography (CT) scans, and so on [8,9,10,11,12]. Additionally, CNN has been highly successful in computer vision (CV), speech recognition, natural language processing (NLP), and audio recognition. Moreover, a neural network (NN) is a sequence of algorithms that identify relationships in a series of data that are exactly the same as the operation of the human brain [13]. For image processing and pattern recognition, this technique is highly successful. It takes an image as the input and constructs a model that processes the image to extract the features and identify a pattern. With these patterns, CNN can recognize similarities of new input images [14,15]. This technique is highly successful due to its adaptability, simple structure, the low complexity of the network model, and reduced training parameters. COVID-19 detection using CNN became a powerful method after the first cases became a global pandemic [16]. One study found outstanding CNN-based research using CT and X-ray images to identify and classify COVID-19. These are not an alternative to actual testing methods, although the CNN technique has had remarkable success [17]. This technique is highly beneficial when used with actual testing methods, but before commercial use, further research and development must be conducted. 

This study designs a sine cosine optimization with deep learning-based disease detection and classification (SCODL-DDC) for COVID-19 on chest X-ray (CXR) images. The proposed SCODL-DDC technique examines the CXR images to identify and classify the presence of COVID-19. In particular, the SCODL-DDC technique uses the EfficientNet model for feature vector generation and its hyperparameters can be adjusted by the SCO algorithm. Furthermore, the quantum neural network (QNN) model can be employed for accurate COVID-19 classification process. Finally, an equilibrium optimizer (EO) is used for optimum parameter selection in the QNN model. The experimental outcomes of the SCODL-DDC method are tested on a benchmark CXR dataset. 

The rest of the paper is organized as follows: Section 2 provides the related works and Section 3 offers the proposed model. Then, Section 4 gives the result analysis and Section 5 concludes the paper.

## 2. Related Works

In [18], the authors introduced an LW-CORONet method, which is a lightweight CNN method that encompasses a sequence of convolution layers, pooling layers, the two fully connected (FC) layers, and the rectified linear unit (ReLU). This method facilitates the extraction of useful features from CXR images with five learnable layers. Gupta and Bajaj [19] devised a robust structure utilizing deep learning (DL)-based techniques and chest CT-scan images for automatic screening of COVID-19. In this study, two pre-trained DL methods, DarkNet19 and MobileNetV2, publicly accessible CT-scan image data, and a lightweight DL technique were employed for automatic screening of COVID-19. In [20], a new technique was modelled to enrich the classification and screening of COVID-19 patients related to their CXR images. In this method, by integrating the conventional data augmentation methods with the generative adversarial networks (GANs), the author not only solved the data limitation issue, but also allowed a deeper extraction of attributes through the implementation of different filter banks such as the Gabor filters, Sobel, and Laplacian of Gaussian (LoG).

Ravi et al. [21] devised largescale learning techniques, including a stacked ensemble Meta classifier and a DL-related feature fusion technique for the classification of COVID-19. The extraction of attributes from global average pooling (penultimate layer) belonging to EfficientNet-related pre-trained methods was completed, and utilizing kernel principal component analysis (PCA), the dimensionality of extracted features was minimalized. Then, to join the features of different mined features, a method called the fusion approach was used. Lastly, for classification purposes, a stacked ensemble meta-classifier-based approach was used. In [22], the authors proposed an innovative Bayesian optimization-based CNN technique for recognizing CXR imageries. This method has two major elements. The first element uses CNN to learn and extract deep features. The second element is a Bayesian-based optimizer that can be exploited for tuning the CNN hyperparameter as per an objective function.

Mansour et al. [23] devised a new, unsupervised DL-based variational autoencoder (UDL-VAE) method for the recognition and classification of COVID-19. To enhance the image quality, the proposed method included an adaptive Wiener filtering (AWF)-related preprocessing method. Additionally, InceptionV4 included the Adagrad method utilized as a feature extractor. For classification, an unsupervised VAE technique was implemented. A set of experiments was conducted to identify the potential outcome of the UDL-VAE method and to validate its detection accuracy. Shankar and Perumal [24] presented a fusion model handcrafted with DL features (FM-HCF-DLF) as a method for the classification and diagnosis of COVID-19. The FM method integrated the handcrafted feature with a local binary pattern (LBP) and DL features, and used the CNN-based InceptionV3 method. To additionally enhance the InceptionV3 model performance, a learning rate scheduler utilizing the Adam optimizer (AO) was implemented. Finally, to effectuate the classification process, a multilayer perceptron (MLP) was used.

In [25], a new artificial neural network (ANN), convolution capsule network (CapsNet), for recognition of COVID-19 was presented by utilizing CXR images with CapsNets. The presented method was intended to offer correct and quick diagnostics for COVID-19 with binary and multi-class classifications. Almalki et al. [26] examined a new approach, CoVIRNet (COVID Inception-ResNet method), which exploits the CXRs for automatically diagnosing people with COVID-19. The presented method has various inception residual blocks which provide data-utilizing various depths, mapping features at various scales, with several layers. These features can be concatenated at every presented classification block, utilizing the average-pooling layer, and concatenated features can be passed to the FC layer. Shorfuzzaman et al. [27] presented a new CNN-based DL fusion structure utilizing the transfer learning (TL) method, but parameters (weights) in various methods were integrated as a single method for extracting features in images that were provided to the custom classifier to forecast. Bekhet et al. [28] examined an AI-based approach for primary COVID-19 analysis in CXR images utilizing medical experience and deep CNNs. Finally, a DL technique was generated carefully and fine-tuned for achieving maximal performance in COVID-19 recognition.

Although several ML and DL models for COVID-19 classification are available in the literature, there is still a need to enhance the classification performance. Due to the continuous deepening of the model, the number of parameters of DL models also increases quickly, which results in model overfitting. At the same time, different hyperparameters have a significant impact on the efficiency of the CNN model. Particularly, hyperparameters, such as epoch count, batch size, and learning rate selection, are essential to attain effectual outcomes. Since the trial-and-error method for hyperparameter tuning is a tedious and erroneous process, metaheuristic algorithms can be applied. Therefore, in this work, we employed SCO and EO algorithms used for the parameter selection of the EfficientNet and QNN models, respectively.

## 3. The Proposed Model

**Figure 1:**
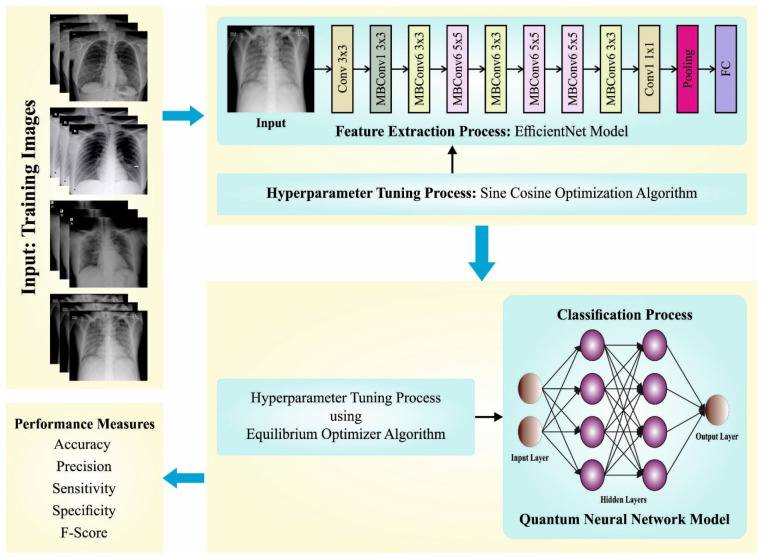
Overall procedure of the SCODL-DDC approach.

In this study, we introduced a new SCODL-DDC algorithm for automated and accurate COVID-19 classification models on CXR images. The proposed SCODL-DDC technique examines the CXR images to identify and classify the presence of COVID-19. To accomplish this, it encompasses the EfficientNet feature extractor, SCO-based hyperparameter tuning, QNN-based classification, and EO-based parameter tuning. The overall procedure of the SCODL-DDC algorithm is illustrated in Figure 1.

### 3.1. Feature Extraction Using EfficientNet

In this work, the SCODL-DDC technique used the EfficientNet model for feature vector generation. Tan and Le recently studied the connection between the depth and width of the CNN and proposed a powerful approach to design a CNN model with few parameters, but a great classification performance [29]. They developed seven models, which they represented as EfficientNetB0 to EfficientNetB7. Together, they were named the EfficientNet CNN model. Once the EfficientNet CNN model was employed in the ImageNet datasets, it was demonstrated that their model outperformed every new model with respect to the number of parameters and Top-1 accuracy. New technology for CNN scaling is the basis for the EfficientNet family. It exploits a powerful and straightforward compound coefficient. Unlike classical techniques that scale features of organizations, such as goal, width, and profundity, EfficientNet scales every aspect with an appropriate set of scaling coefficients. Scaling individual aspects acts on model implementation but adjusting each organization component with respect to the available resources works on implementation.

EfficientNet is considerably smaller than other models with comparable accuracy to ImageNet. As found in the Keras application, for example, the ResNet50 model has 23,534,592 boundaries. It still needs to meet the expectation of the small EfficientNet (named EfficientNetB0) model, which has 5,330,564 boundaries. An effective model is proposed on the basis of EfficientNetB3 CNN since it strikes a proper balance between computational power and accuracy. The mobile inverted-bottleneck convolution (MBConv) is a building block of the EfficientNet family. The concept of the MobileNet model is the basis for MBConv. The fundamental concept is to apply depthwise separable convolution that involves a depthwise and a pointwise convolutional layer. The two basic concepts, (1) Linear bottlenecks and (2) Residual connections that were inverted are taken from MobileNetV2: 

The EfficientNet family starts with its stem which is common to every eight models and the last layers. After the stem, there exist seven blocks. In addition, this block has different sub-blocks, and the number rises as they progress from EfficientNetB0 to EfficientNetB7. The total number of layers in EfficientNetB0 is 237, whereas the total number of layers in EfficientNetB7 is 813. The next module is the basis for the initial sub-block of the seven major blocks, except the first. Module Three brings together each sub-block through a skip connection. The skip connection in the initial sub-block is merged with Module Four. Module Five connects with each sub-block through a skip fashion to the one before it. Lastly, a sub-block is generated by combining the module with being used in a specific way in the block.

### 3.2. Hyperparameter Tuning

The hyperparameters of the EfficientNet model can be chosen by the SCO algorithm at this stage. In 2016, Seyedali Mirjalili proposed an SCO, a newly established metaheuristic technique that is used to resolve real-time engineering problems [30]. The mathematical model is based primarily on the sine and cosine rules, and SCO changes many initial random solutions to the best possible region of convergence. Furthermore, SCO uses variables that pose adaptive and random behaviors for relocating a bad solution into the best possible region with ease. For this reason, the algorithm was extensively used. The SCO uses two fundamental processes for search: population and local search strategies. Both strategies are responsible for local exploitation and global exploration. The broad applicability of SCO is due to its hassle-free, adaptive, and simple structure. The SCO has been hybridized with different approaches for robust mathematical optimization. Due to this feature, SCO is widely employed to resolve various optimization problems, such as feature selection, scheduling, economic power dispatch planning, power energy, classification, and benchmark functions.

The candidate solution in SCO was expressed as a matrix without losing any generality:

(1) $$X=\left\{\begin{array}{lll} x_{\left(1,1\right)} & x_{\left(1,2\right)} & x_{\left(1,d\right)}\\ x_{\left(2,1\right)} & x_{\left(2,2\right)} & x_{\left(2,d\right)}\\ \vdots & \vdots & \vdots \\ x_{\left(n,1\right)} & x_{\left(n,2\right)} & x_{\left(n,d\right)} \end{array}\right\}$$

In Equation (1), the row vector can be signified for the subsequent entries $X_{n}=[x_{n,1},x_{n,2},\ldots ,x_{n,.d}].$ Like other optimization techniques, the initialization technique has a matrix formation that consists of size (N × d). Furthermore, the location updating expression depends on the assessment of trigonometric function with an encoding step. The sine and cosine functions are used for updating the position, as follows:

(2) $$Y_{i}^{t+1}=Y_{i}^{t}+r_{1}\times sin\left(r_{2}\right)\times \left|r_{3}j_{i}^{t}-Y_{i}^{t}\right|$$

(3) $$Y_{i}^{t+1}=Y_{i}^{t}+r_{1}\times cos\left(r_{2}\right)\times \left|r_{3}j_{i}^{t}-Y_{i}^{t}\right|$$

Here, $Y_{i}^{t}$ denotes the location of the present solution in i−th parameter at t−th iteration. Furthermore, randomness is added by incorporating $r_{1}/r_{2}/r_{3}$. Equations (2) and (3) characterize the location update to attain the desired destination point at i−th parameter. Furthermore, the updated formula uses the absolute value of the difference as follows:

(4) $$Y_{i}^{t+1}=\{Y_{i}^{t}+m_{1}\times sin(r_{2})\times \left|r_{3}j_{i}^{t}-Y_{i}^{t}\right|$$

(5) $$Y_{i}^{t+1}=\{Y_{i}^{t}+m_{1}\times cos(r_{2})\times \left|r_{3}j_{i}^{t}-Y_{i}^{t}\right|$$

In Equations (4) and (5), $j_{i}^{\tau }$ represents the local optimum solution, random integer within [0, 2π]. Therefore, the search direction can be aggregated to the global optimum solution by incorporating the sine and cosine rules. $r_{3}$ is the uniform distribution random number between [0, 2]. Furthermore, the bridging is provided with the help of a monotonically decreasing linear function, $m_{1}$. This number decreases with the increment in iterative count increasing.

(6) $$m_{1}=a-t*\frac{a}{T_{max}}$$

In Equation (6), a denotes the constant, t and $T_{max}$ denote the present iteration and max iteration count which determines the ending condition for the optimization algorithm. As mentioned before, the balancing between the exploitation and exploration stages can be performed by using *m*_1_. The success of optimization algorithm greatly depends on these parameters.

Fitness selection is a crucial factor in the SCO approach. Solution encoding is exploited for assessing the aptitude (goodness) of candidate solution. Now, the accuracy value is the main condition utilized for designing a fitness function

(7) $$Fitness=max\left(P\right)$$

(8) $$P=\frac{TP}{TP+FP}$$

where the true positive value can be represented as TP and the false positive value can be denoted as FP.

### 3.3. Optimal QNN-Based Classification

For COVID-19 classification, the QNN model is used. The architecture of the quantum neuron model is based on quantum logic gate, involving the reverse rotation part, phase rotation part, output part, input part, and aggregation part [31]. The working process of QNN is given as follows:

Step 1: let $|x_{i}\rangle =(cost_{i},sint_{i}{)}^{T}$, as well as define the qubit phase rotation gate using Equation (9):

(9) $$R\left(\theta \right)=\left(\begin{array}{cc} \cos\theta & -\sin\theta \\ \sin\theta & \cos\theta \end{array}\right)$$

Next, with the aggregation, the equation becomes

(10) $$\sum_{i=1}^{n}R(\theta_{i})|x_{i}\rangle =[cos\theta sin\theta {]}^{T},$$

where $\theta =arg(\sum_{i=1}^{n}R(\theta_{i})|x_{i}\rangle )=argtan(\sum_{i=1}^{n}sin(t_{i}+\theta_{i})/\sum_{i=1}^{n}cos(t_{i}+\theta_{i}))$.

Step 2: the outcomes of Equation (7) make the reverse rotation operation by the controlled-NOT gate:

(11) $$U\left(\gamma \right)=\left(\begin{array}{cc} \cos\left(f\left(\gamma \right)\frac{\pi }{2}-2\theta_{0}\right) & -\sin\left(f\left(\gamma \right)\frac{\pi }{2}-2\theta_{0}\right)\\ \sin\left(f\left(\gamma \right)\frac{\pi }{2}-2\theta_{0}\right) & \cos\left(f\left(\gamma \right)\frac{\pi }{2}-2\theta_{0}\right) \end{array}\right),$$

where f denotes the sigmoid function as

(12) $$U(\gamma )\sum_{i=1}^{n}R(\theta_{j})|x_{j}\rangle =[cos\left(\frac{\pi }{2}f\left(\gamma \right)-\theta \right)sin\left(\frac{\pi }{2}f\left(\gamma \right)-\theta \right){]}^{T}$$

Thus, the relationships between the input and output of the quantum neuron models are defined below:

(13) $$y=sin\left(\frac{\pi }{2}f\left(\gamma \right)-\theta \right)=sin\left(\frac{\pi }{2}f(\gamma )-arg(\sum_{i=1}^{n}R(\theta_{j})|x_{j}\rangle )\right).$$

The ship-steering controller design is created by using the quantum neuron model. The hidden layer (HL), output layer, and input layer are the three layers of the presented method. The QNN is used in the layer that is between the input layers and HLs; there is p conventional neuron in the output layer, n quantum neuron in the input layer, and m quantum neuron in the HL. Taking the qubit as the transfer function of the hidden layer, then the output of the QNN can be expressed as follows:

(14) $$y_{k}=g(\sum_{j=1}^{m}w_{jk}h_{j})=g\left(\sum_{j=1}^{m}w_{jk}sin\left(\frac{\pi }{2}f\left(\gamma_{j}\right)-arg\left(\sum_{i=1}^{m}R\left(\theta_{ij}\right)|x_{i}\rangle \right)\right)\right),$$

where i=1,2,…,n;j=1,2,…,m; and k=1,2,…,p.

**Figure 2:**
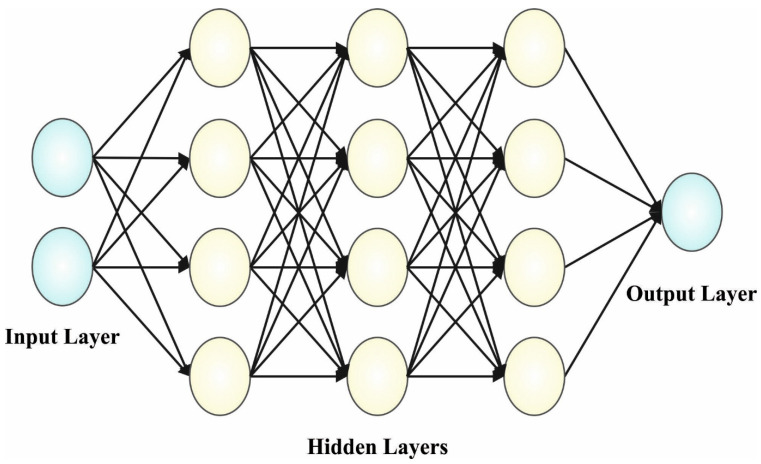
Architecture of the QNN.

In Equation (14), $y_{k}$ represents the output of QNN, $|x_{i}\rangle$ denotes the input variable, $h_{j}$ denotes the output of HL, $w_{jk}$ indicates the network weight for the output layer and HL, and $R(\theta_{ij})$ shows the quantum rotation gate between the input and the HLs for updating the qubits. Figure 2 illustrates the architecture of the QNN method.

At the final stage, the EO algorithm is used for parameter optimization purposes. Using the dynamic mass balance, the main concept of single objective EO was established [32]. These characteristics could maintain the balance between detection and exploitation and the ability to maintain flexibility amongst every individual solution. At first, EO exploits a certain group, whereas every particle describes the vector focus that has a solution to the problems.

(15) $$Y_{j}^{initial}=lb+rand_{j}\left(ub-lb\right),j=0,1,2,3,...,n$$

where $Y_{j}^{initial}$ represents the vector focus on $j^{th}$ particles, ub and lb characterize the upper and lower limits of the problem, $rand_{j}$ specifies the randomly generated number within [0,1], and n indicates the number of particles. In the exploration and exploitation approaches, the five equilibrium candidates help EO. The first four candidates search for optimum exploration. However, the fifth candidate with average values searches for modification from exploitation.

(16) $${\vec{C}}_{eq,pool}=\left\{{\vec{C}}_{eq\left(1\right)},{\vec{C}}_{eq\left(2\right)},{\vec{C}}_{eq\left(3\right)},{\vec{C}}_{eq\left(4\right)},{\vec{C}}_{eq\left(ave\right)}\right\}$$

The upgrade of concentration assists EO in equally balancing exploration and exploitation:

(17) $$\vec{F}=e^{-\vec{\lambda }(t-t_{0})}$$

where $\vec{\lambda }$ specifies the randomly generated number within [0, 1], and t minimizes as the iteration amount increases.

(18) $$t=(1-\frac{It}{{Max}_{-}it}{)}^{\left(a_{2}\frac{It}{{Max}_{-}it}\right)}$$

where It and ${Max}_{-}it$ represent the present and maximal amount of the iteration, and $a_{2}$ denotes the constant control of the ability for exploitation. Another variable, $a_{1}$, was exploited for enhancing exploration and exploitation:

(19) $$t=\frac{1}{\vec{\lambda }}ln\left(-a_{1}sign\left(\vec{r}-O.5\right)\left[1-e^{\vec{-\lambda }t}\right]\right)+t$$

The generation rate can be represented by as G rises exploitation:

(20) $$\vec{G}={\vec{G}}_{0}e^{-\vec{l}(t-t_{0})}$$

where $\vec{l}$ indicates the randomly generated number [0,1], and the initial generation rate is denoted as ${\vec{G}}_{0}$:

(21) $${\vec{G}}_{0}=G\vec{C}P\left({\vec{C}}_{eq}-\vec{\lambda }\vec{C}\right)$$

(22) $$G\vec{C}P=\left\{\begin{array}{l} 0.5r_{1},r_{2}\geq GP\\ 0,r_{2}<GP \end{array}\right.$$

where t$r_{1}$ and $r_{2}$ represent the randomly generated number ranges within [0,1]. The vector $\vec{GC}P$ signifies the control variable which controls the generation rate implemented for the upgrading stage.

(23) $$\vec{C}=\vec{C}+\left(\vec{C}-{\vec{C}}_{eq}\right).\vec{F}+\frac{\vec{C}}{\vec{\lambda }V}\left(1-\vec{F}\right)$$

The value of V corresponds to 1. The EO method not only derives a fitness function to accomplish superior accuracy of the classification but also delineates a positive integer to characterize the remarkable performance of the solution candidate. The reduction in the classification error rate is regarded as a fitness function.

(24) $$\begin{array}{c} fitness\left(x_{i}\right)=ClassifierErrorRate\left(x_{i}\right)\\ =\frac{number\;of\;misclassified\;samples}{Total\;number\;of\;samples}*100 \end{array}$$

## 4. Results and Discussion

The proposed model is simulated using Python 3.7 on PC i5-8600k, GeForce 1050Ti 4 GB, 16 GB RAM, 250 GB SSD, and 1 TB HDD. The QNN model is implemented in Python using a quantum computing library called Qiskit. Qiskit supports several different backends, including simulators and real quantum devices. The parameter settings are given as follows: learning rate: 0.01, dropout: 0.5, batch size: 5, and number of epochs: 50.

**Figure 3:**
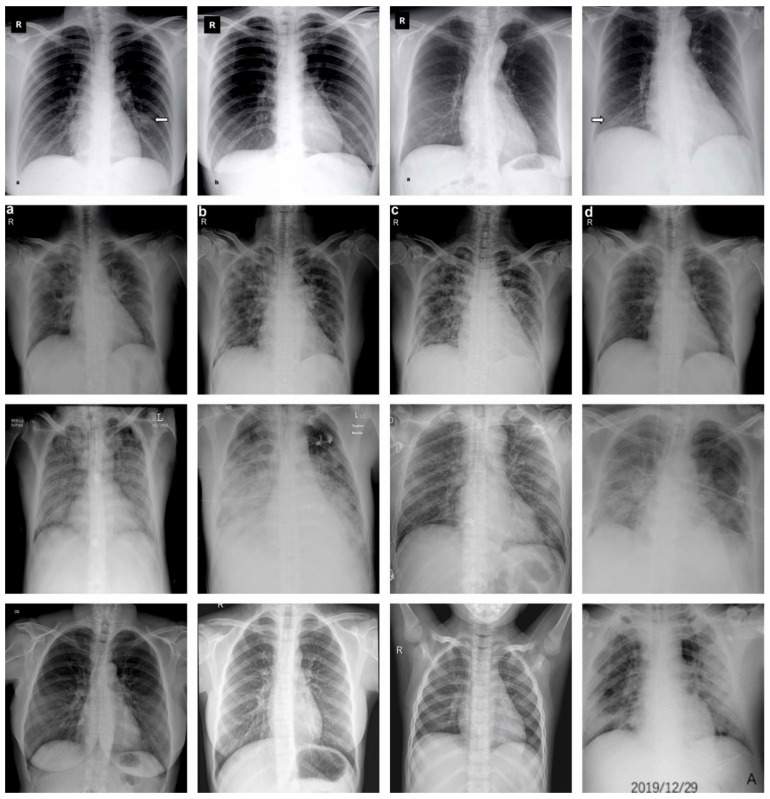
Sample images (A: airways; B: breathing; C: circulation; D: disability; R: Rotation; Arrow Mark: Opacities).

In this section, the experimental validation of the SCODL-DDC technique is tested on the CXR image dataset [33], comprising 305 samples with different classes as defined in Table 1. Figure 3 represents the sample images.

**Figure 4:**
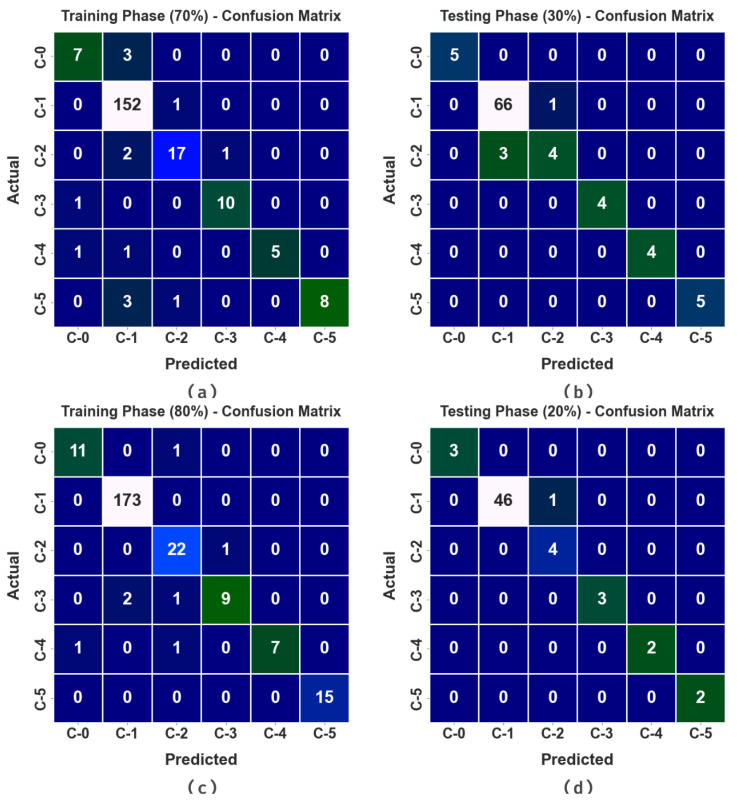
Confusion matrices of the SCODL-DDC approach (**a**,**b**) TRP/TSP of 70:30 and (**c**,**d**) TRP/TSP of 80:20.

In Figure 4, the COVID-19 detection results from the SCODL-DDC technique are demonstrated in the form of confusion matrices. This figure revealed that the SCODL-DDC technique identifies different classes efficaciously.

In Table 2, an overall classification outcome of the SCODL-DDC technique is studied under varying sizes of TRP and TSP. These results showcase the enhanced results of the SCODL-DDC technique in all cases. For instance, on 70% of TRP, the SCODL-DDC technique attains an average $accu_{y}$ of 97.81%, a $prec_{n}$ of 92.10%, a $sens_{y}$ of 80.56%, a $spec_{y}$ of 97.08%, and an $F_{score}$ of 85.32%. Meanwhile, on 30% of TSP, the SCODL-DDC technique attains an average $accu_{y}$ of 98.55%, a $prec_{n}$ of 95.94%, a $sens_{y}$ of 92.61%, a $spec_{y}$ of 97.80%, and an $F_{score}$ of 93.95%. Eventually, on 80% of TRP, the SCODL-DDC method achieves an average $accu_{y}$ of 99.04%, a $prec_{n}$ of 94.75%, a $sens_{y}$ of 90.02%, a $spec_{y}$ of 99.16%, and an $F_{score}$ of 92.01%.

**Figure 5:**
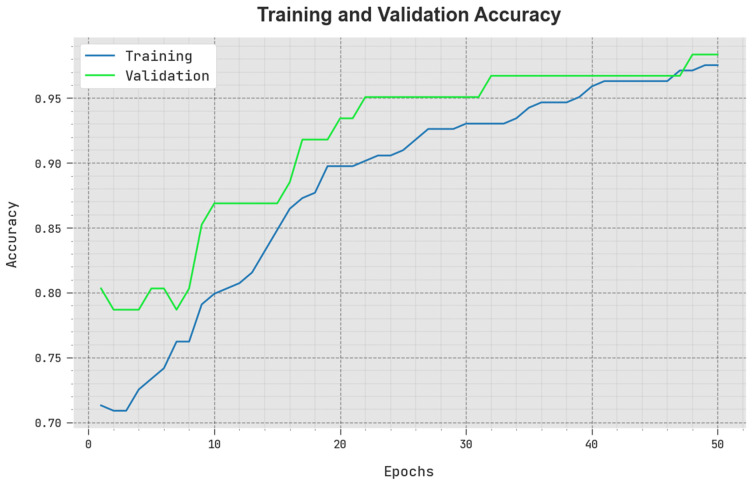
TACY and VACY outcome of the SCODL-DDC approach.

The TACY and VACY of the SCODL-DDC method are inspected on COVID-19 detection performance in Figure 5. The figure reveals that the SCODL-DDC approach has demonstrated superior performance with maximum values of TACY and VACY. It can be observed that the SCODL-DDC method has attained higher TACY outcomes.

**Figure 6:**
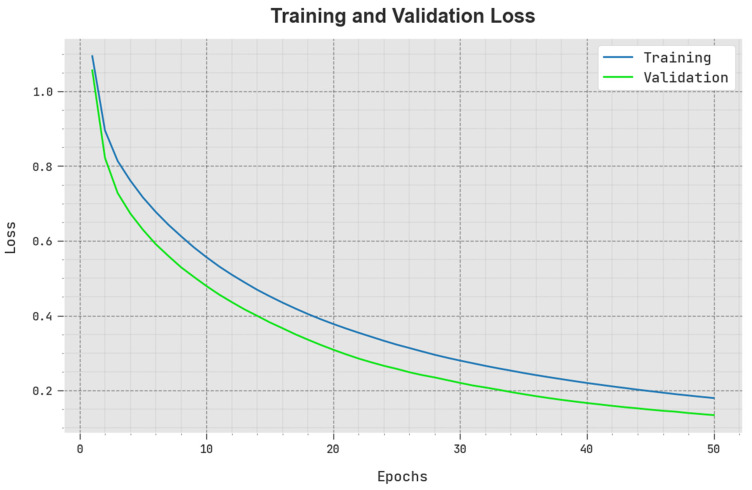
TLOS and VLOS outcome of SCODL-DDC method.

The TLOS and VLOS of the SCODL-DDC method are tested on COVID-19 detection performance in Figure 6. The figure implies that the SCODL-DDC approach has illustrated superior performance with the lowest values of TLOS and VLOS. It is demonstrated that the SCODL-DDC method has resulted in minimum VLOS outcomes.

**Figure 7:**
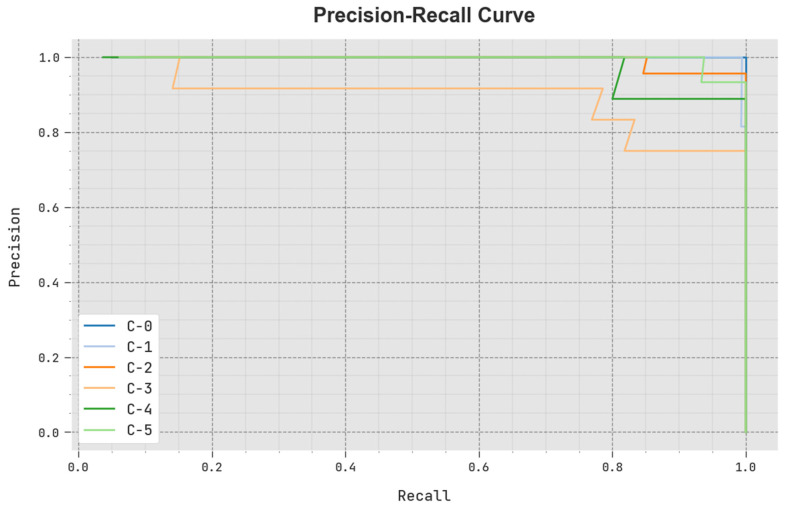
Precision-recall outcome of the SCODL-DDC approach.

A clear precision-recall analysis of the SCODL-DDC technique under a test database is portrayed in Figure 7. The figure indicates that the SCODL-DDC approach results in improved values of precision-recall values in all classes.

**Figure 8:**
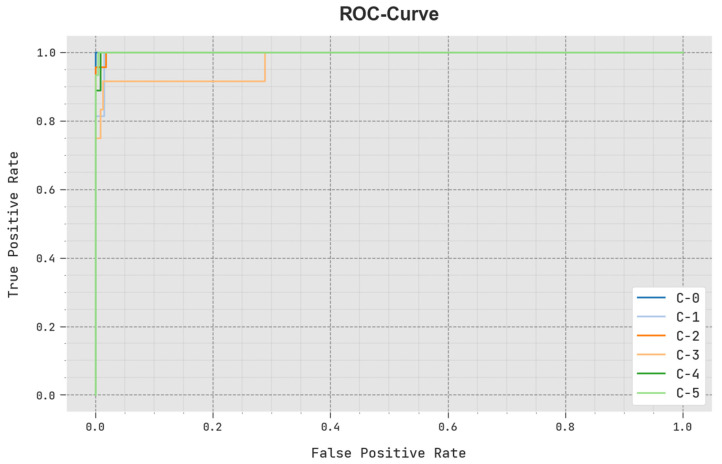
ROC curve outcome of the SCODL-DDC approach.

A brief ROC examination of the SCODL-DDC approach under a test database is depicted in Figure 8. The outcomes represented by the SCODL-DDC approach show its ability in classifying various classes.

The comparison study of the SCODL-DDC technique with other COVID-19 classifiers is given in Table 3 [34]. Based on $sens_{y}$, the SCODL-DDC technique reaches an increasing $sens_{y}$ of 99.65% while the fusion, Inception v3, ResNet-50, VGG-16, DLS-SCD, DLA-CVD, AD-TLCNN, and FM-HCF-DLF models attain a decreasing $sens_{y}$ of 92.86%, 94.26%, 88.17%, 86.57%, 86.21%, 87.22%, 99.46%, and 93.59%, respectively. Meanwhile, based on $spec_{y}$, the SCODL-DDC method obtained a maximum $spec_{y}$ of 99.71% whereas the fusion, InceptionV3, ResNet50, VGG16, DLS-SCD, DLA-CVD, AD-TLCNN, and FM-HCF-DLF techniques obtain a decreasing $spec_{y}$ of 98.45%, 97.68%, 97.85%, 97.80%, 86.66%, 87.59%, 97.18%, and 94.77%, respectively. Based on $accu_{y}$, the SCODL-DDC method reaches an increasing $accu_{y}$ of 99.45% while the fusion, Inception v3, ResNet-50, VGG-16, DLS-SCD, DLA-CVD, AD-TLCNN, and FM-HCF-DLF methods accomplish a decreasing $accu_{y}$ of 98.97%, 97.65%, 97.08%, 96.59%, 86.44%, 89.69%, 95.07%, and 94.71%, respectively. Meanwhile, based on $F_{score}$, the SCODL-DDC method obtains an increasing $F_{score}$ of 97.97% whereas the fusion, InceptionV3, ResNet50, VGG16, DLS-SCD, DLA-CVD, AD-TLCNN, and FM-HCF-DLF models accomplish a decreasing $F_{score}$ of 93.16%, 90.43%, 84.07%, 83.26%, 86.01%, 76.36%, 92.33%, and 93.60%, respectively. These results support the enhanced COVID-19 classification outcomes of the SCODL-DDC technique.

## 5. Conclusions

In this study, we introduced a new SCODL-DDC algorithm for automated and accurate COVID-19 classification algorithms on CXR images. The proposed SCODL-DDC technique examines the CXR images to identify and classify the presence of COVID-19. To accomplish this, it encompasses the EfficientNet feature extractor, SCO-based hyperparameter tuning, QNN-based classification, and EO-based parameter tuning. Moreover, the SCODL-DDC technique uses the EfficientNet model for feature vector generation and its hyperparameters can be adjusted by the SCO algorithm. Lastly, the EO algorithm with the QNN model is employed for an accurate COVID-19 classification process. The experimental results of the SCODL-DDC technique were tested on benchmark CXR datasets and the outcomes exhibited superior performance over other approaches. In the future, feature fusion with ensemble voting classifiers can be designed to enhance the performance of the SCODL-DDC algorithm.

## Figures and Tables

**Table 1:** Details of the database.

Label	Class	Number of Images
C-0	ARDS	15
C-1	COVID-19	220
C-2	No Finding	27
C-3	Pneumocystis	15
C-4	SARS	11
C-5	Streptococcus	17
**Total Number of Images**		**305**

**Table 2:** Classifier outcome of the SCODL-DDC approach on varying sizes of TRP/TSP.

Labels	$Accu_{y}$	$Prec_{n}$	$Sens_{y}$	$Spec_{y}$	$F_{score}$
**Training Phase (70%)**					
C-0	97.65	77.78	70.00	99.01	73.68
C-1	95.31	94.41	99.35	85.00	96.82
C-2	97.65	89.47	85.00	98.96	87.18
C-3	99.06	90.91	90.91	99.50	90.91
C-4	99.06	100.00	71.43	100.00	83.33
C-5	98.12	100.00	66.67	100.00	80.00
**Average**	**97.81**	**92.10**	**80.56**	**97.08**	**85.32**
**Testing Phase (30%)**					
C-0	100.00	100.00	100.00	100.00	100.00
C-1	95.65	95.65	98.51	88.00	97.06
C-2	95.65	80.00	57.14	98.82	66.67
C-3	100.00	100.00	100.00	100.00	100.00
C-4	100.00	100.00	100.00	100.00	100.00
C-5	100.00	100.00	100.00	100.00	100.00
**Average**	**98.55**	**95.94**	**92.61**	**97.80**	**93.95**
**Training Phase (80%)**					
C-0	99.18	91.67	91.67	99.57	91.67
C-1	99.18	98.86	100.00	97.18	99.43
C-2	98.36	88.00	95.65	98.64	91.67
C-3	98.36	90.00	75.00	99.57	81.82
C-4	99.18	100.00	77.78	100.00	87.50
C-5	100.00	100.00	100.00	100.00	100.00
**Average**	**99.04**	**94.75**	**90.02**	**99.16**	**92.01**
**Testing Phase (20%)**					
C-0	100.00	100.00	100.00	100.00	100.00
C-1	98.36	100.00	97.87	100.00	98.92
C-2	98.36	80.00	100.00	98.25	88.89
C-3	100.00	100.00	100.00	100.00	100.00
C-4	100.00	100.00	100.00	100.00	100.00
C-5	100.00	100.00	100.00	100.00	100.00
**Average**	**99.45**	**96.67**	**99.65**	**99.71**	**97.97**

**Table 3:** Comparative outcome of the SCODL-DDC method with other models [34].

Methods	Accuracy	Sensitivity	Specificity	F-Score
SCODL-DDC	99.45	99.65	99.71	97.97
Fusion Model	98.97	92.86	98.45	93.16
Inception V3 Model	97.65	94.26	97.68	90.43
ResNet-50 Model	97.08	88.17	97.85	84.07
VGG-16 Model	96.59	86.57	97.80	83.26
DLS-SCD	86.44	86.21	86.66	86.01
DLA-CVD	89.69	87.22	87.59	76.36
AD-TLCNN	95.07	99.46	97.18	92.33
FM-HCF-DLF	94.71	93.59	94.77	93.60

## Data Availability

Data sharing is not applicable to this article as no datasets were generated during the current study.
